# Racial/Ethnic Differences in Long-COVID-Associated Symptoms among Pediatrics Population: Findings from Difference-in-differences Analyses in RECOVER Program

**DOI:** 10.21203/rs.3.rs-4151744/v1

**Published:** 2024-03-28

**Authors:** Yong Chen, Dazheng Zhang, Bingyu Zhang, Qiong Wu, Ting Zhou, Jiayi Tong, Yiwen Lu, Jiajie Chen, Huiyuan Wang, Deena Chisolm, Ravi Jhaveri, Rachel Kenney, Russel Rothman, Suchitra Rao, David Williams, Mady Hornig, Jeffrey Morris, Christopher Forrest

**Affiliations:** University of Pennsylvania; University of Pennsylvania; University of Pennsylvania; University of Pennsylvania; University of Pennsylvania; University of Pennsylvania; University of Pennsylvania; University of Pennsylvania; University of Pennsylvania; Abigail Wexner Research Institute Nationwide Children’s Hospital; Ann & Robert H. Lurie Children’s Hospital of Chicago; New York University Grossman School of Medicine; Vanderbilt University Medical Center; University of Colorado School of Medicine and Children’s Hospital; University of Michigan; Columbia University Mailman School of Public Health; University of Pennsylvania; Children’s Hospital of Philadelphia

## Abstract

Racial/ethnic differences are associated with the potential symptoms and conditions of post-acute sequelae SARS-CoV-2 infection (PASC) in adults. These differences may exist among children and warrant further exploration. We conducted a retrospective cohort study for children and adolescents under the age of 21 from the thirteen institutions in the RECOVER Initiative. The cohort is 225,723 patients with SARS-CoV-2 infection or COVID-19 diagnosis and 677,448 patients without SARS-CoV-2 infection or COVID-19 diagnosis between March 2020 and October 2022. The study compared minor racial/ethnic groups to Non-Hispanic White (NHW) individuals, stratified by severity during the acute phase of COVID-19. Within the severe group, Asian American/Pacific Islanders (AAPI) had a higher prevalence of fever/chills and respiratory symptoms, Hispanic patients showed greater hair loss prevalence in severe COVID-19 cases, while Non-Hispanic Black (NHB) patients had fewer skin symptoms in comparison to NHW patients. Within the non-severe group, AAPI patients had increased POTS/dysautonomia and respiratory symptoms, and NHB patients showed more cognitive symptoms than NHW patients. In conclusion, racial/ethnic differences related to COVID-19 exist among specific PASC symptoms and conditions in pediatrics, and these differences are associated with the severity of illness during acute COVID-19.

## INTRODUCTION

The post-acute sequelae SARS-CoV-2 infection (PASC) has emerged as a significant concern^1–3^, particularly among young individuals with a previous diagnosis of COVID-19 ^4–7^. Defined by the World Health Organization (WHO) as the persistence of at least one physical symptom for 12 weeks following initial testing without an alternative diagnosis and expanded by the National Institutes of Health (NIH) to include ongoing, relapsing, or new symptoms four or more weeks post-acute infection, PASC potentially affects a significant proportion of COVID-19 survivors^8–10^. In pediatric populations, recent studies show that potential PASC symptoms and conditions tend to be systematic and/or syndromic, with higher incidence conditions such as loss of taste/smell, myocarditis, and symptoms associated with cold-like illness occurring in patients after the acute phase of COVID-19^11^. The estimated prevalence of potential PASC symptoms and conditions in the pediatric population ranges from 23–45% among those previously infected by SARS-CoV-2^12–14^, depending on study designs and PASC definitions. These findings highlight the urgent need for further research and comprehensive support to address the prevalence of PASC in pediatric populations.

Prior investigations into potential racial/ethnic differences in PASC among adults have unearthed important findings. For example, the Centers for Disease Control and Prevention (CDC) have shown variations in PASC’s impact based on race/ethnicity^15^. A study by Khullar et al.^16^ reported that Non-Hispanic Black (NHB) individuals exhibited a higher incidence of new PASC symptoms and conditions compared to Non-Hispanic White (NHW) patients, a difference more pronounced in hospitalized than in non-hospitalized patients. These findings suggest the existence of potential racial/ethnic differences in PASC among adults. Importantly, it is crucial to note that race and ethnicity are social constructs rather than biological ones ^17,18^. Concurrently, research indicates children’s likelihood of testing positive for COVID-19 correlates with their race/ethnicity ^19–23^. NHB, Hispanic, and multi-racial children exhibited higher rates of COVID-19 positivity compared to their NHW counterparts, indicating differences in infection rates across different racial/ethnic groups^24^. However, limited research to date has addressed potential racial/ethnic differences in PASC among children and adolescents, making it a pressing area of study. Therefore, our study aimed to quantify such racial/ethnic differences by conducting an association study involving children and adolescents, to determine if the observed patterns are consistent with the findings from studies conducted among adults.

Examining health outcomes through the lens of racial/ethnic differences in the context of COVID-19 runs the risk of pre-existing racial/ethnic differences being either overshadowed or underestimated. To address this, we employed a difference-in-differences approach to discern the shifts in racial/ethnic differences before and after COVID-19. As existing research has focused predominantly on adult populations, the clinical evidence regarding these differences among the pediatric population also remains uncertain. Our investigation centered on a pediatric cohort from the RECOVER electronic health records (EHR) database across thirteen institutions. To the best of our knowledge, this is the first and the largest study for the pediatric population with the longest follow-up that investigates the racial difference in PASC symptoms and conditions attributable to SARS-CoV-2 infection. This study also included the Asian American/Pacific Islanders (AAPI) population, which was notably absent in prior research. Our study aims to answer several key unaddressed questions. First, we sought to compare the incidence of potential PASC symptoms and conditions among COVID-19-positive patients with the incidence among those without documented SARS-CoV-2 infection. Second, we aimed to ascertain whether COVID-19 status correlates with any racial/ethnic differences across potential PASC symptoms and conditions. More importantly, we quantified the racial/ethnic differences linked with COVID-19 by carefully accounting for pre-infection disparities through the application of difference-in-differences analyses. We also applied a novel negative control method to adjust for the impacts of potential unmeasured confounders. By addressing these questions, our objective is to shed light on the relationship between COVID-19, racial/ethnic differences, and potential PASC symptoms and conditions.

## RESULTS

The study involved 225,723 children and adolescents with COVID-19 and 677,448 without COVID-19. The COVID-19-positive cohort comprised 109,022 NHW patients (48.3%), 45,823 NHB patients (20.3%), 60,012 Hispanic patients (26.6%), and 10,866 AAPI patients (4.8%) with SARS-CoV-2 infection from March 2020 to October 2022. Of these, 50.2% were female. Baseline comorbidities by race/ethnicity and severity are in Table 1. The COVID-19-negative cohort included 365,113 NHW patients (53.9%), 130,465 NHB patients (19.3%), 149,686 Hispanic patients (22.1%), and 32,184 AAPI patients (4.8%) without SARS-CoV-2 infection or MIS-C diagnosis.

Incidence of PASC symptoms and conditions for COVID-19 positive and negative patients

Table 2 presents the incidence for 24 potential PASC symptoms and conditions in the COVID-19 positive cohort compared with the COVID-19 negative cohort, stratified by acute COVID-19 severity status. The data in Table 2 reveal that the incidence rates of all listed PASC symptoms and conditions were significantly increased in COVID-19-positive patients as compared with the COVID-19-negative group during the follow-up period. For example, the incidence of respiratory signs and symptoms for COVID-19-positive patients was 9.68% while the incidence was 7.25% for the COVID-19-negative group (P < 0.001). Moreover, the incidence of the potential PASC symptoms and conditions in the severe group was increased compared to the incidence of these symptoms and conditions within the non-severe COVID-19 patient group. s

### Racial/ethnic differences in PASC symptoms and conditions

After achieving the balance of SMD (**Section S8**), Fig. 3 shows the racial/ethnic difference attributable to COVID-19 in potential PASC symptoms and conditions by the severity of COVID-19. Overall, we found moderate evidence of an increase in composite outcomes, i.e., at least one condition and any of the syndromic conditions, after SARS-CoV-2 infection among the AAPI group in both severe and non-severe COVID-19 group, but there was no strong evidence of increased racial differences among Hispanic and Non-Hispanic Black groups.

For patients with severe COVID-19, AAPI patients showed a higher increase in any of the conditions (RR 1.24, 95% confidence interval (CI) 1.04 to 1.49, P = 0.019) and any of syndromic conditions (RR 1.22, 95% CI 1.01 to 1.47, P = 0.042) compared to NHW after SARS-CoV-2 infection. Hispanic patients showed no increase in any of the conditions (RR 0.99, 95% CI 0.91 to 1.08, P = 0.804), and NHB patients showed a minor decrease in any of the conditions (RR 0.93, 95% CI 0.85 to 1.24, P = 0.147) as compared to NHW patients. For patients with non-severe COVID-19, AAPI patients showed a higher increase in any of the conditions (RR 1.08, 95% CI 1.01 to 1.14, P = 0.015) and any of syndromic conditions (RR 1.08, 95% CI 1.01 to 1.08, P = 0.017) compared to NHW. Hispanic patients showed almost no increase in any of the conditions (RR 1.01, 95% CI 0.98 to 1.04, P = 0.498), and NHB patients also showed almost no decrease in any of the conditions (RR 0.99, 95% CI 0.89 to 1.11, P = 0.915) as compared to NHW patients.

However, there exist statistically significant differences among all minority groups across several PASC symptoms and conditions after SARS-CoV-2 infection. For example, for patients with severe COVID-19, the increased prevalence of hair loss among Hispanic patients was greater (RR 2.62, 95% CI 1.06 to 6.49, P = 0.038) than the increased prevalence among NHW patients. The corresponding increase in the prevalence of fever and chills among AAPI was greater (RR 1.41, 95% CI 1.01 to 1.97, P = 0.045). NHB patients had a smaller increase in skin symptoms (RR 0.74, 95% CI 0.58 to 0.96, P = 0.021) than NHW patients. For patients with non-severe COVID-19, AAPI patients had a greater increase concerning POTS/dysautonomia (RR 1.57, 95% CI 1.02 to 2.40, P = 0.037) and respiratory signs and symptoms (RR 1.11, 95% CI 1.00 to 1.23, P = 0.036) compared to NHW patients. NHB patients had a greater increase in cognitive functions (1.25, 95% CI 1.01 to 1.55, P = 0.037) than NHW patients.

Furthermore, we observed a differential increase by racial/ethnic group within both severe and non-severe groups. These racial/ethnic differences varied depending upon the severity of the acute phase of COVID-19 as well as the specific potential PASC symptoms and conditions being analyzed. For example, among the severe group, the differential increase in abdominal pain was more pronounced for all three minority groups compared to those in the non-severe category.

### Sensitivity analysis

Figure S2 showed the results of the negative control outcome experiments and estimated systematic error, such as the unmeasured confounder bias. **Figure S3** showed the racial/ethnic differences after SARS-CoV-2 infection stratified by severity of COVID-19, using standard regression models. Among COVID-19 patients within the severe group, NHB patients showed a greater incidence in any of the conditions (RR 1.16, 95% CI 1.02 to 1.32, P = 0.024) and any syndromic conditions (RR 1.14, 95% CI 1.00 to 1.30, P = 0.042) as compared to NHW patients. Hispanic patients also showed a greater incidence in any of the conditions (RR 1.12, 95% CI 0.99 to 1.27, P = 0.075) as compared to NHW patients.

Specifically, among COVID-19 patients with severe illness during the acute infection, Hispanic individuals exhibited a greater incidence of respiratory signs and symptoms (RR: 1.16, 95% CI 1.02 to 1.33, P = 0.024) and hair loss (RR: 1.84, 95% CI 1.02 to 3.31, P = 0.043) as compared with the NHW patient group. NHB had a greater incidence of respiratory signs and symptoms (RR: 1.19, 95% CI 1.03 to 1.36, P = 0.015) and heart disease (RR: 1.48, 95% CI 1.06 to 2.07, P = 0.022), but a lower incidence of arrhythmias (RR: 0.73, 95% CI 0.57 to 0.94, P = 0.013) and headache (RR: 0.66, 95% CI 0.48 to 0.93, P = 0.016) compared with the NHW group.

Among those with non-severe acute COVID-19, Hispanic patients displayed a higher incidence of myocarditis (RR 4.28, 95% CI 1.53 to 11.98, P = 0.006) and abnormal liver enzyme (RR 2.06, 95% CI 1.08 to 3.94, P = 0.029) compared with NHW patients. Meanwhile, AAPI patients demonstrated a greater incidence of hair loss (RR 3.32, 95% CI 1.43 to 7.72, P = 0.005) compared with the NHW patient group.

These findings revealed that our difference-in-differences approach identified fewer racial/ethnic differences compared to standard regression models. It is worth noting that the difference-in-differences approach adjusted for the baseline racial/ethnic difference before the SARS-CoV-2 infection, a step that a standard regression analysis failed to take into consideration. Consequently, some of the observed racial/ethnic differences with prior work might not be attributed to COVID-19. Nevertheless, given its adjustment for baseline racial/ethnic differences, the difference-in-differences approach holds greater robustness.

In the analysis including only those patients identified based on positive SARS-CoV-2 PCR or antigen testing (**Section S4**), differences among severe patients were diminished among some potential PASC symptoms and conditions, while among the non-severe patients, the differences that we identified were consistent in both sets of analyses. To account for the potential bias stemming from limited hospital capacity during the initial COVID-19 period, we performed a secondary analysis excluding COVID-19 patients from the first wave of the pandemic (March to May 2020). This exclusion did not significantly alter the results, as demonstrated in **Section S5**. **Section S6** shows the results of subgroup analysis by age group. shows the results of stratified analysis by virus variants.

## DISCUSSION

We examined racial/ethnic variations in long-term consequences of documented SARS-CoV-2 infection across thirteen health institutions in the RECOVER study. Our analysis revealed a higher incidence of potential Post-Acute Sequelae of SARS-CoV-2 (PASC) symptoms in COVID-19-positive patients, with differences attributed to severity. Notably, NHB patients showed a smaller increase in skin symptoms compared to NHW patients, consistent with previous findings in adults. This observation aligns with the findings reported for the adult population by Khullar et al.^30^. After accounting for pre-existing differences and confounder bias, moderate evidence suggested greater differences attributable to COVID-19 for AAPI compared to NHW, while no strong evidence indicated disparities in composite outcomes for NHB or Hispanic populations compared to NHW.

Our study has multiple strengths. First, we used propensity score matching methods instead of linear regression models in our adjustment of the confounders, which helped us reduce the non-linear effects of the confounders^31^. Second, we accounted for the pre-infection racial/ethnic difference in long-term COVID-19-related symptoms and conditions. This enabled us to quantify racial/ethnic differences attributable to COVID-19 in the PASC symptoms and conditions and to control any pre-infection differences in these health issues. Third, we used negative control outcomes to calibrate the systematic bias, which is powerful in controlling the unmeasured confounders.

Our study has several research directions that warrant future investigations. First, socioeconomic differences due to race/ethnicity may exacerbate racial/ethnic differences in potential PASC symptoms and conditions, thereby acting as a mediator effect in the causal pathway between race/ethnicity and clinical outcomes. Such influences have been suggested as risk factors for acute COVID-19 by Chisolm and colleagues in a RECOVER EHR study ^32^. Future research on PASC outcomes is of interest to study such mediation effects.

Secondly, health-seeking behavior or healthcare access is an important consideration^33^. It is possible that certain minority racial groups have more limited access to care and associated medical records and that this contributes to potential bias in the observed racial/ethnic differences. Related issues were recently described by Nasir et al.^34^ for the ascertainment of PASC symptoms and conditions in adult populations through EHR^34^.

Thirdly, confounding poses a significant bias threat in observational studies. To address this, we extensively adjusted for potential confounders using a propensity-score-based matching method and difference-in-differences analyses. We employed negative control outcomes to reduce the residual bias, such as unmeasured confounder bias. Additionally, EHR data completeness issues may lead to misclassification and loss-to-follow-up bias. Some attempts have been made to mitigate the impacts of these biases^35–38^. The analysis used a combined set of patients, but potential race/ethnicity bias may vary between outpatients and inpatients. Addressing these issues can help improve the reliability of evidence generated from these investigations.

In summary, we rigorously quantified the racial/ethnic differences in potential PASC symptoms and conditions and the impact of SARS-CoV-2 infection on these differences. The impact of COVID-19 varied across racial/ethnic groups, severity of acute COVID-19, and different PASC symptoms and conditions.

## METHODS

### DATA SOURCES

This retrospective cohort study is part of the NIH Researching COVID-19 to Enhance Recovery (RECOVER) Initiative (https://recovercovid.org/), which aims to learn about the long-term effects of COVID-19. The data were contributed by thirteen sites. Participating institutions in this study included: Children’s Hospital of Philadelphia, Cincinnati Children’s Hospital Medical Center, Children’s Hospital of Colorado, Ann & Robert H. Lurie Children’s Hospital of Chicago, Nationwide Children’s Hospital, Nemours Children’s Health System (in Delaware and Florida), Duke University, University of Iowa Healthcare, University of Michigan, University of Missouri, OCHIN, University of California, San Francisco, and Vanderbilt University Medical Center.

### COHORT CONSTRUCTION

**Figure 1:**
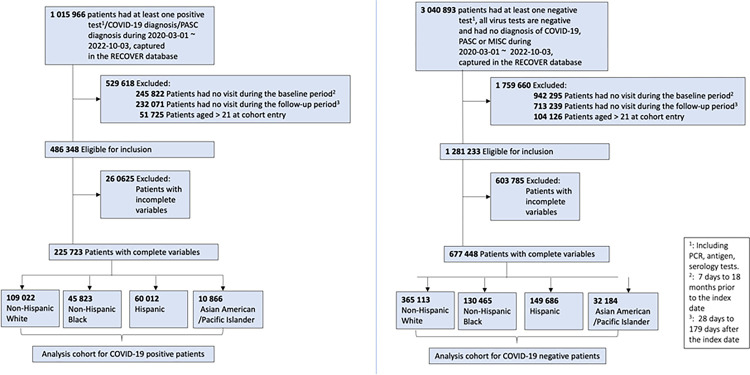
Selection of participants for both COVID-19 positive and negative patients. The “patients with complete variables” refer to patients with complete records of BMI, race/ethnicity, and vaccine records.

We conducted a retrospective study from March 1, 2020, to October 3, 2022, with at least 6 months of follow-up time. We included patients under the age of 21 who had at least one visit within 18 months to 7 days before the index date (defined as the baseline period) and at least one encounter within 28 days and 179 days after the index date (defined as the follow-up period). For COVID-19-positive patients, we included the patients who had positive polymerase-chain-reaction (PCR), serology, or antigen tests or diagnoses of COVID-19, or post-acute sequelae of SARS-CoV-2 (PASC), which we defined as documented SARS-CoV-2 infection. The index date for these patients was defined as the first time of SARS-CoV-2 infection. For COVID-19-negative patients, we included patients who had neither a documented SARS-CoV-2 infection nor a diagnosis of multisystem inflammatory syndrome in children (MISC) within the same study period, and who had at least one negative COVID-19 test result. A random negative test was chosen as the index date for COVID-19-negative patients. The selection of participants for both COVID-19 positive and negative patients in real-world data is summarized in Fig. 1.

### DEFINING OUTCOMES

Our definition of potential PASC symptoms and conditions included 24 symptoms and conditions as shown in Rao et al.^11^, including abdominal pain, abnormal liver enzyme, acute kidney injury, acute respiratory distress syndrome, arrhythmias, cardiovascular signs and symptoms, changes in taste and smell, chest pain, cognitive functions, fatigue and malaise, fever and chills, fluid and electrolyte, generalized pain, hair loss, headache, heart disease, mental health disorders, musculoskeletal pain, myocarditis, myositis, Postural Orthostatic Tachycardia Syndrome (POTS) or dysautonomia, respiratory signs and symptoms, skin symptoms, and thrombophlebitis and thromboembolism. Systematic and syndromic conditions related to PASC were grouped by the 24 potential PASC symptoms and conditions.

### PATIENT CHARACTERISTICS

The primary exposure was race/ethnicity, categorized into NHW, NHB, Hispanic, and AAPI. Other/unknown racial/ethnic groups were classified as missing or race/ethnicity not listed above and were excluded due to small sample sizes. Various patient characteristics were considered as confounders, such as age at the cohort entry date (< 5, 5–12, 12–21), gender (female, male), cohort entry month (from March 2020 to October 2022), site indicators, obesity (obese, non-obese), a chronic condition indicator as defined by the Pediatric Medical Complexity Algorithm (PMCA, no chronic condition, non-complex chronic condition, and complex chronic condition)^25^, healthcare visits (inpatient, outpatient, and emergency department visits), medications (0, 1, 2, ≥ 3), negative tests (0, 1, 2, ≥ 3), vaccine doses (0, 1, ≥ 2), and immunization duration during the baseline period (no vaccine, < 4 months, ≥ 4 months). The severity of COVID-19 at the cohort entry date was stratified into the following categories: asymptomatic, mild (symptomatic), moderate (involving moderately severe COVID-19-related conditions like gastroenteritis, dehydration, and pneumonia), and severe (comprising unstable COVID-19-related conditions, ICU admission, or mechanical ventilation)^26^. In this paper, we categorized patients exhibiting either asymptomatic or mild symptoms as belonging to the “non-severe” group, while all other patients were classified as part of the “severe” group.

### STATISTICAL METHODS

We calculated the incidence of potential PASC symptoms and conditions in both COVID-19 positive and negative cohorts stratified by severity. For each PASC symptom or condition, we calculated its incidence by dividing the number of patients who experienced the symptom or condition during the follow-up period but not at baseline by the total number of patients. To quantify the racial/ethnic differences in the potential PASC symptoms and conditions, we use relative risk (RR) as the comparative measure. The RR is known to be a collapsible measure, where collapsibility^27^ refers to the measure of association conditional on some factors that remain consistent with the marginal measure collapsed over strata^28^.

**Figure 2:**
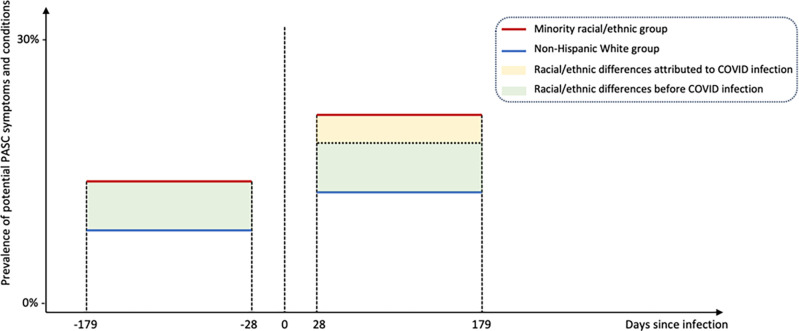
Illustration of difference-in-differences analysis for disentangling racial/ethnic differences related to COVID-19 infections in potential PASC symptoms and conditions from the pre-infection observed racial/ethnic differences.

**Figure 3:**
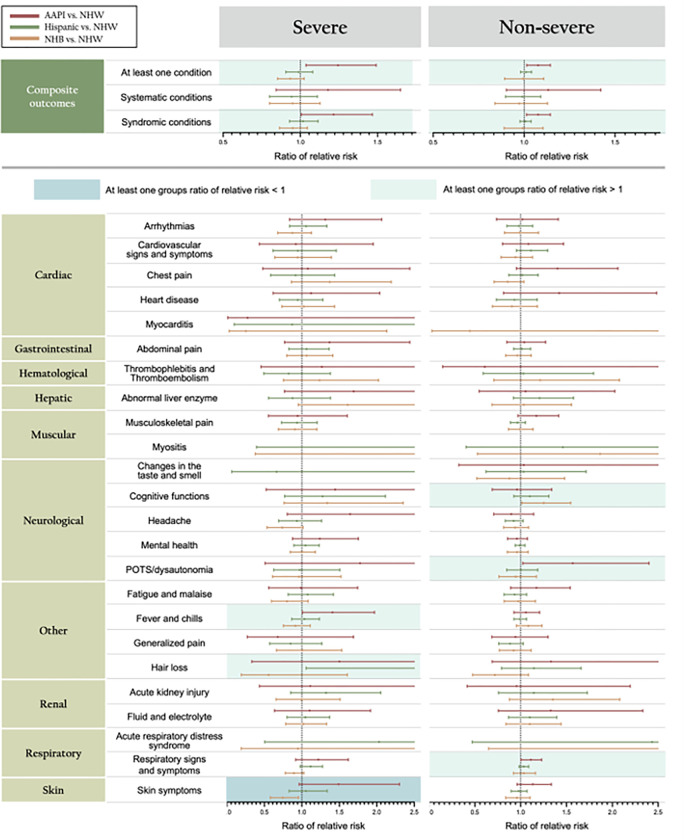
Racial/Ethnic Differences that are attributable to COVID-19 estimated from the difference-in-differences (DiD) analyses for prevalence of potential PASC symptoms and conditions among COVID-19-positive patients, by race/ethnicity and severity status.

To eliminate the impact of potential measured confounders, we used a propensity score matching technique with the covariates detailed in the patient characteristics section. The propensity score is calculated by the logistic regression model fitted by regressing the racial/ethnic groups on the covariates. We performed this matching separately for minority racial/ethnic groups (NHB, Hispanic, and AAPI), each stratified by severity status, compared with the NHW group. After performing the matching, we assessed the standardized mean difference (SMD) between each covariate value for different racial/ethnic groups, with a difference of 0.1 or less indicating an acceptable balance^29^. Subsequently, we quantified the differential increase in the prevalence of potential PASC symptoms and conditions across different racial/ethnic groups by the difference-in-difference method. A Poisson regression model was fitted by regressing the potential PASC symptoms and conditions on racial/ethnic groups, SARS-CoV-2 infection status, and their interaction terms. Figure 2 provides a visual representation of the difference-in-difference method used to estimate racial/ethnic differences in the increased prevalence of potential PASC symptoms and conditions related to COVID-19.

### SENSITIVITY ANALYSES

We conducted a list of sensitivity analyses to examine the robustness of our findings. First, to evaluate how different statistical methods might influence the analytical results, we used an alternative approach, multivariate regression analyses, with RR as the comparative measure. Specifically, we considered the incidence of potential PASC symptoms and conditions as outcomes, while controlling for the same confounders that were used in the matching process within the difference-in-differences analysis. Second, we conducted analyses for COVID-19 patients identified only by positive SARS-CoV-2 PCR or antigen tests, because the recorded date of COVID-19 diagnosis may not accurately reflect the actual infection date. Third, we conducted analyses excluding patients whose index dates fell within the first wave of COVID-19 (March to May 2020) due to limited SARS-CoV-2 testing availability during this period. Additionally, our sensitivity analysis featured stratification by a set of age group strata (< 5, 5–12, 12–21), differing from the ones previously specified, and by estimated time frames corresponding to dominant virus variants (pre-Delta, Delta, Omicron).

### ADJUSTMENT FOR UNMEASURED CONFOUNDERS

While we used propensity score matching to account for the measured confounders and difference-in-differences analyses to address pre-infection racial/ethnic differences, the results can still be impacted by unmeasured confounder bias. To mitigate such bias, we collected 31 negative control outcomes, as prespecified by pediatric physicians, that should not exhibit racial/ethnic differences due to COVID-19. By using these negative control outcomes, the study was able to calibrate the residual bias from unmeasured and systematic sources. A comprehensive explanation of our statistical methods can be found in **Section S1** of the Supplementary Materials.

## Figures and Tables

**Table 1:** Baseline characteristics of COVID-19 positive patients, by race/ethnicity and severity status.

	*Severe*				*Non-severe*			
	NHW (N = 9,140)	AAPI (N = 590)	Hispanic (N = 3,786)	NHB (N = 3,346)	NHW (N = 99,882)	AAPI (N = 10,276)	Hispanic (N = 56,226)	NHB (N = 42,477)
**Age categories (%)**								
< 1 years	1601 (17.5%)	122 (20.7%)	767 (20.3%)	733 (21.9%)	9655 (9.7%)	965 (9.4%)	6154 (10.9%)	4686 (11.0%)
1 to < 5 years	2811 (30.8%)	197 (33.4%)	1174 (31.0%)	910 (27.2%)	22018 (22.0%)	2802 (27.3%)	11892 (21.2%)	9147 (21.5%)
5 to < 12 years	1893 (20.7%)	133 (22.5%)	773 (20.4%)	568 (17.0%)	27547 (27.6%)	3191 (31.1%)	17205 (30.6%)	12991 (30.6%)
12 to < 16 years	1243 (13.6%)	60 (10.2%)	489 (12.9%)	482 (14.4%)	18276 (18.3%)	1433 (13.9%)	10430 (18.6%)	7721 (18.2%)
16 to < 21 years	1592 (17.4%)	78 (13.2%)	583 (15.4%)	653 (19.5%)	22386 (22.4%)	1885 (18.3%)	10545 (18.8%)	7932 (18.7%)
**Gender**								
Female	4208 (46.0%)	255 (43.2%)	1728 (45.6%)	1580 (47.2%)	50682 (50.7%)	4968 (48.3%)	28412 (50.5%)	21408 (50.4%)
Male	4932 (54.0%)	335 (56.8%)	2058 (54.4%)	1766 (52.8%)	49200 (49.3%)	5308 (51.7%)	27814 (49.5%)	21069 (49.6%)
**Hospital**								
A	1459 (16.0%)	37 (6.3%)	154 (4.1%)	389 (11.6%)	14323 (14.3%)	530 (5.2%)	1226 (2.2%)	6069 (14.3%)
B	1113 (12.2%)	95 (16.1%)	317 (8.4%)	646 (19.3%)	24713 (24.7%)	1960 (19.1%)	4289 (7.6%)	9949 (23.4%)
C	1031 (11.3%)	57 (9.7%)	801 (21.2%)	163 (4.9%)	4415 (4.4%)	242 (2.4%)	3880 (6.9%)	848 (2.0%)
D	390 (4.3%)	29 (4.9%)	159 (4.2%)	257 (7.7%)	4647 (4.7%)	471 (4.6%)	2245 (4.0%)	4443 (10.5%)
E	420 (4.6%)	13 (2.2%)	60 (1.6%)	48 (1.4%)	7581 (7.6%)	322 (3.1%)	1094 (1.9%)	1047 (2.5%)
F	712 (7.8%)	71 (12.0%)	595 (15.7%)	250 (7.5%)	1907 (1.9%)	239 (2.3%)	1833 (3.3%)	661 (1.6%)
G	754 (8.2%)	28 (4.7%)	92 (2.4%)	153 (4.6%)	7547 (7.6%)	742 (7.2%)	768 (1.4%)	1314 (3.1%)
H	232 (2.5%)	1 (0.2%)	16 (0.4%)	35 (1.0%)	4529 (4.5%)	109 (1.1%)	283 (0.5%)	710 (1.7%)
I	1156 (12.6%)	85 (14.4%)	183 (4.8%)	517 (15.5%)	8530 (8.5%)	1098 (10.7%)	1801 (3.2%)	5844 (13.8%)
J	882 (9.6%)	33 (5.6%)	643 (17.0%)	501 (15.0%)	8173 (8.2%)	526 (5.1%)	5824 (10.4%)	3404 (8.0%)
K	105 (1.1%)	25 (4.2%)	273 (7.2%)	64 (1.9%)	6783 (6.8%)	2568 (25.0%)	28574 (50.8%)	5440 (12.8%)
L	182 (2.0%)	88 (14.9%)	320 (8.5%)	100 (3.0%)	1942 (1.9%)	1151 (11.2%)	3170 (5.6%)	1057 (2.5%)
M	704 (7.7%)	28 (4.7%)	173 (4.6%)	223 (6.7%)	4792 (4.8%)	318 (3.1%)	1239 (2.2%)	1691 (4.0%)
**Entry time**								
03/2020–06/2020	136 (1.5%)	17 (2.9%)	163 (4.3%)	122 (3.6%)	907 (0.9%)	190 (1.8%)	1935 (3.4%)	891 (2.1%)
07/2020–10/2020	241 (2.6%)	19 (3.2%)	166 (4.4%)	108 (3.2%)	5454 (5.5%)	433 (4.2%)	4967 (8.8%)	1760 (4.1%)
11/2020–02/2021	753 (8.2%)	47 (8.0%)	346 (9.1%)	291 (8.7%)	16011 (16.0%)	1240 (12.1%)	10089 (17.9%)	5262 (12.4%)
03/2021–06/2021	720 (7.9%)	32 (5.4%)	277 (7.3%)	309 (9.2%)	7916 (7.9%)	445 (4.3%)	3007 (5.3%)	4056 (9.5%)
07/2021–10/2021	1063 (11.6%)	37 (6.3%)	394 (10.4%)	485 (14.5%)	13868 (13.9%)	818 (8.0%)	6095 (10.8%)	6477 (15.2%)
11/2021–02/2022	3050 (33.4%)	193 (32.7%)	1320 (34.9%)	1136 (34.0%)	33830 (33.9%)	3746 (36.5%)	19028 (33.8%)	16290 (38.4%)
03/2022–06/2022	1662 (18.2%)	120 (20.3%)	577 (15.2%)	447 (13.4%)	12030 (12.0%)	1951 (19.0%)	4939 (8.8%)	3739 (8.8%)
07/2022–10/2022	1515 (16.6%)	125 (21.2%)	543 (14.3%)	448 (13.4%)	9866 (9.9%)	1453 (14.1%)	6166 (11.0%)	4002 (9.4%)
**Obesity**								
0	6055 (66.2%)	422 (71.5%)	2230 (58.9%)	1926 (57.6%)	62750 (62.8%)	6141 (59.8%)	21525 (38.3%)	21711 (51.1%)
1	3085 (33.8%)	168 (28.5%)	1556 (41.1%)	1420 (42.4%)	37132 (37.2%)	4135 (40.2%)	34701 (61.7%)	20766 (48.9%)
**PMCA**								
0	4333 (47.4%)	311 (52.7%)	1938 (51.2%)	1552 (46.4%)	67424 (67.5%)	7594 (73.9%)	41294 (73.4%)	27868 (65.6%)
1	1435 (15.7%)	79 (13.4%)	534 (14.1%)	524 (15.7%)	18614 (18.6%)	1564 (15.2%)	8820 (15.7%)	8692 (20.5%)
2	3372 (36.9%)	200 (33.9%)	1314 (34.7%)	1270 (38.0%)	13844 (13.9%)	1118 (10.9%)	6112 (10.9%)	5917 (13.9%)
**Negative tests prior entry**								
0	6038 (66.1%)	407 (69.0%)	2671 (70.5%)	2301 (68.8%)	73195 (73.3%)	7908 (77.0%)	45852 (81.5%)	32592 (76.7%)
1	1445 (15.8%)	97 (16.4%)	521 (13.8%)	497 (14.9%)	15999 (16.0%)	1440 (14.0%)	6650 (11.8%)	6233 (14.7%)
2	641 (7.0%)	32 (5.4%)	225 (5.9%)	238 (7.1%)	5875 (5.9%)	519 (5.1%)	2169 (3.9%)	2146 (5.1%)
>=3	1016 (11.1%)	54 (9.2%)	369 (9.7%)	310 (9.3%)	4813 (4.8%)	409 (4.0%)	1555 (2.8%)	1506 (3.5%)
**Vaccine dosage**								
0	7860 (86.0%)	469 (79.5%)	3347 (88.4%)	3015 (90.1%)	82136 (82.2%)	7256 (70.6%)	46760 (83.2%)	37252 (87.7%)
1	240 (2.6%)	22 (3.7%)	80 (2.1%)	71 (2.1%)	2878 (2.9%)	474 (4.6%)	1667 (3.0%)	1159 (2.7%)
>=2	1040 (11.4%)	99 (16.8%)	359 (9.5%)	260 (7.8%)	14868 (14.9%)	2546 (24.8%)	7799 (13.9%)	4066 (9.6%)

**Table 2:** Raw incidence (%) of potential PASC symptoms and conditions comparing COVID-19 positive andnegative patients.

	COVID-19 positive (%)			COVID-19 negative (%)		
	*All (N = 225,723)*	*Severe (N = 16,862)*	*Non-severe (N = 208,861)*	*All (N = 677,448)*	*Severe (N = 143,592)*	*Non-severe (N = 533,856)*
*At least one condition*	26.86	34.17	26.42	21.44	19.8	21.84
*Systematic conditions*	2.69	7.46	2.37	1.87	2.93	1.61
*Syndromic conditions*	26.15	32.48	25.75	20.83	18.77	21.35
*Abdominal pain*	3.04	3.66	2.99	2.27	2.15	2.31
*Abnormal liver enzyme*	0.32	1.17	0.25	0.24	0.43	0.19
*Acute kidney injury*	0.22	1.32	0.13	0.15	0.42	0.08
*Acute respiratory distress syndrome*	0.03	0.24	0.01	0.01	0.03	0.01
*Arrhythmias*	1.43	4.41	1.21	0.95	1.56	0.79
*Cardiovascular signs and symptoms*	1.16	1.69	1.12	0.88	0.86	0.89
*Changes in the taste and smell*	0.16	0.08	0.17	0.04	0.02	0.05
*Chest pain*	1.39	1.69	1.37	0.74	0.68	0.75
*Cognitive functions*	0.68	1	0.66	0.66	0.62	0.68
*Fatigue and malaise*	1.74	3.22	1.63	1.3	1.74	1.18
*Fever and chills*	5.67	9.15	5.41	3.73	3.31	3.84
*Fluid and electrolyte*	0.56	3.33	0.36	0.39	0.92	0.24
*Generalized pain*	1.26	1.78	1.22	0.91	1.02	0.88
*Hair loss*	0.24	0.48	0.22	0.14	0.14	0.14
*Headache*	2.2	2.33	2.19	1.51	1.23	1.59
*Heart disease*	0.37	1.67	0.27	0.28	0.62	0.2
*Mental health*	6.32	8.04	6.19	5.45	5.05	5.55
*Musculoskeletal pain*	3.53	4.03	3.5	2.79	2.72	2.81
*Myocarditis*	0.03	0.17	0.02	0.01	0.02	0
*Myositis*	0.02	0.08	0.02	0.02	0.02	0.01
*POTS/dysautonomia*	1.03	1.34	1.01	0.71	0.72	0.71
*Respiratory signs and symptoms*	9.68	15.02	9.31	7.25	6.77	7.38
*Skin symptoms*	3.62	4.99	3.51	2.7	2.66	2.71
*Thrombophlebitis and thromboembolism*	0.13	1.02	0.06	0.11	0.33	0.05

Note:

* indicates no evidence of statistical significance from the two-sample proportion test (see the Supplementary Materials)

Red Symptom: COVID-19 positive higher incidence

Blue Symptom: COVID-19 positive lower incidence (see the Supplementary Materials)

## Data Availability

The data can be shared with the request to the RECOVER initiative.
